# Susceptibility of Chicken Embryos, Sheep, Cattle, Pigs, and Chickens to Zika Virus Infection

**DOI:** 10.3389/fvets.2020.00023

**Published:** 2020-02-05

**Authors:** Aruna Ambagala, Thang Truong, Colleen Cottam-Birt, Yohannes Berhane, Volker Gerdts, Uladzimir Karniychuk, David Safronetz, Shawn Babiuk

**Affiliations:** ^1^Canadian Food Inspection Agency, National Centre for Foreign Animal Disease, Winnipeg, MB, Canada; ^2^Department of Animal Science, University of Manitoba, Winnipeg, MB, Canada; ^3^Vaccine and Infectious Disease Organization-International Vaccine Centre (VIDO-InterVac), University of Saskatchewan, Saskatoon, SK, Canada; ^4^Department of Veterinary Microbiology, University of Saskatchewan, Saskatoon, SK, Canada; ^5^Zoonotic Diseases and Special Pathogens, Public Health Agency of Canada, Winnipeg, MB, Canada; ^6^Department of Immunology, University of Manitoba, Winnipeg, MB, Canada

**Keywords:** Zika virus, embryo, tissue tropism, susceptibility, chicken

## Abstract

The susceptibility of sheep, cattle, pigs, chickens and chicken embryos to Zika virus infection was evaluated by experimental inoculation with Zika virus Thailand strain isolated from a Canadian traveler in 2013. The inoculated animals did not develop any clinical signs of disease nor evidence of Zika virus replication in peripheral blood, cerebrospinal fluid and tissues including brain and spinal cord assessed by real-time RT-PCR. Sera were also negative for Zika virus antibodies by Zika virus neutralization assays as well as Zika virus immunoperoxidase staining of Zika infected Vero cells. Chicken embryos were inoculated by different routes including yolk sac (4 day old embryos), chorioallantoic membrane (8 day old embryos), amniotic fluid (8 day old embryos) and intravenous routes (12 day old embryos). Virus replication in chicken embryos was observed in the brain and body tissues following intravenous (IV), yolk sac (YS), chorioallantoic membrane (CAM), and amniotic fluid (AF) inoculation routes. The highest mortality was observed in embryos inoculated via yolk sac. The dead embryos showed diffuse muscular hemorrhages. The yolk sac inoculated chicken embryos showed delayed hatching and displayed neurological signs immediately after hatching. These studies demonstrate that 8 week old sheep, 6 month old cattle, 4 week old pigs, and 4 week old chickens are not susceptible to Zika virus infection when inoculated experimentally and therefore unlikely to pose a risk as Zika virus reservoirs. However, chicken embryos are highly susceptible to Zika virus resulting in clinical disease of chicks after hatching. This study demonstrates that Zika virus has a tropism for embryonic tissue and that chicken embryos can be used as a model to study Zika virus replication and pathogenesis.

## Introduction

Zika virus is a Flavivirus originally identified in a febrile Rhesus macaque from the Zika forest region in Africa in 1947 (1). It is spread by *Aedes aegypti*, a mosquito that can also transmit dengue fever, chikungunya and yellow fever viruses. In recent outbreaks in humans, Zika virus caused a mild self-limiting infection with clinical signs of fever, rash, conjunctivitis, arthralgia, and arthritis during an outbreak on Yap Island of the Federated States of Micronesia in 2007 (2). Zika virus then spread to French Polynesia in 2013 (3) where it continued to spread to islands of the Pacific Ocean and then to South America (4). The spread of Zika virus in the Western Hemisphere became a public health emergency due to the link between Zika virus infection and microcephaly in infants (5–7).

Most of the arboviruses depend on nonhuman animal species for maintenance in nature. Many animal species act as reservoirs for these arboviruses, and humans are generally dead-end or accidental hosts to these viruses. Some arboviruses such as dengue virus however, have adapted completely to humans and can be maintained in a mosquito-human-mosquito transmission cycle that does not depend on nonhuman reservoirs. Antibodies to Zika virus have been detected in a number of animal populations including nonhuman primates, farm animals and wild animals (8, 9). In these studies, however, the differentiation between presumably Zika virus specific antibodies and antibodies against other closely related flaviviruses was not comprehensively performed.

Susceptibility of domestic farm animals to experimental Zika virus infection has previously been evaluated in calves, pigs, goats and chickens demonstrating these species as juveniles, young adults or adults are not susceptible (10). However, a different study demonstrated that neonatal pigs were susceptible to Zika virus infection indicating that the age of the animal may be an important factor for Zika virus infection (11). Farm animals, if susceptible to Zika virus infection, may serve as reservoirs for Zika virus increasing the risk of transmission to farm workers, veterinarians and others associated with animal husbandry. Therefore, the main goal of this study was to confirm if sheep, cattle, pigs and chickens are susceptible to Zika virus infection.

Understanding the effect of Zika virus infection on embryonic development is critical. Currently a few animal models using nonhuman primates (12, 13) and mice have been developed (14). These animal models have expanded our knowledge on Zika pathogenesis, however new animal models could provide additional information on Zika virus induced neurological lesions in susceptible hosts. Most current mouse models utilize mice lacking at least one component of the IFN-signaling pathway indicating that IFN signaling is critical for controlling Zika virus replication (15). Although pregnant mouse models using normal mice have been developed in which pups have been demonstrated to be infected with Zika virus (16, 17). In the study reported here, four different domestic animal species were experimentally infected with Zika virus, and they were closely monitored for clinical signs, viral replication and development of humoral immune response to the virus. If Zika virus replicates in these animal species to titers which are high enough to allow for transmission of the virus, they may serve as reservoir for the virus. If they develop clinical signs or pathological lesions similar to that observed in humans, they may be useful animal models to study Zika virus induced pathology.

## Materials and Methods

### Animals, Zika Virus Inoculation, and Sample Collection

Six (8 week old) Rideau Arcott sheep, four (6 months old) Holstein calves, and four (4 week old) Landrace/Large White cross piglets were obtained from a high health status herd operated by a recognized commercial supplier in Manitoba, Canada. Six (2–4 week old) Leghorn chickens were obtained from a specific pathogen free status flock operated by CFIA Ottawa. The age of animals selected was based on availability to procure animals of the youngest age which were not neonates. All animals were housed in separate Biosafety Level 3 animal cubicles at the National Centre for Foreign Animal Disease (Winnipeg, Canada), and were fed a complete balanced diet and water *ad libitum* with a 1 week acclimation period. On day “0,” sheep, cattle and pigs were inoculated intradermally (0.1 ml per site for 5 sites/animal) and intravenous inoculation of 1.0 ml with Zika virus Thailand (18) at 10^6^ TCID50/ml propagated in Vero cells. Chickens were given five subcutaneous inoculations 0.1 ml each and 0.5 ml intravenous inoculation of Zika virus Thailand (10^6^ TCID50/ml). All animal experiments were conducted under the approval of the Canadian Science Centre for Human and Animal Health Animal Care Committee, which follows the guidelines of the Canadian Council on Animal Care. All animals were observed twice daily, with clinical signs recorded throughout the study. Rectal temperatures were measured prior to inoculation for baseline levels and daily from 1 to 21 days post-infection (dpi). Blood and sera, were collected from sheep on days 1, 2, 3, 6, 8, 10, 13, and 21 post-inoculation. Blood and sera were collected from cattle on days 1, 3, 6, 8, 10, 13, and 20 post-inoculation. Blood and sera were collected from pigs on days 3, 5, 6, 7, 10, 12, 14, and 21 post-inoculation. Blood and sera were collected from chickens on days 3, 4, 6, 10, 13, 17, and 21 post-inoculation. Three weeks after inoculation, sheep, cattle, pigs, and chickens were euthanized and necropsies were performed. During the necropsy, animals were assessed for gross pathology cerebrospinal fluid (CSF) as well as tissues including the brain (cortex, midbrain and cerebellum) and spinal cord were collected during the necropsies.

### Egg Inoculations

Zika virus Thailand was inoculated into fertilized specific pathogen free (SPF) chicken eggs (Canadian Food Inspection Agency Fallowfield, Ottawa) at different embryonic stages using different routes of inoculations. The virus was administered using 100 μl inoculations at various doses with the highest doses of 10^7^ PFU/ml, or as 10-fold dilutions. The inoculation procedures were done as follows. For intravenous (IV) inoculations 12 day old embryonated chicken eggs (ECEs) were used. For yolk sac (YS) inoculations 4 day old ECEs were used and for chorioallantoic membrane (CAM) and amniotic fluid (AF) inoculation 8 day old ECEs were used.

Prior to inoculations, eggs were allowed to cool at room temperature for approximately 1 h. For IV inoculation, 27 gauge hypodermic needles were used. For all other routes 1½ inch 23 gauge needles were used. Once inoculated the eggs were incubated at 33°C in a nonrocking incubator for 24 h with 55% relative humidity (RH), and transferred to a nonrocking incubator set at 37°C with 55% RH. Eggs were candled twice daily. All eggs dying within the first 24 h were discarded. The dead eggs were stored at 4°C a minimum of 1 h before the tissues (allantoic fluid, CAM, brain, heart, liver, and eye balls) were harvested. From small embryos, all organs were pooled as body parts.

### Hatching Experiments

Two hatching experiments were conducted, one via CAM inoculation and the second via YS inoculation. For CAM inoculation, a group of thirty six 8 day-old embryonated eggs were inoculated with Zika virus dose of 10^4^ PFU/ml and 36 control eggs were inoculated with sterile phosphate buffered saline (PBS). Eggs were incubated for 7 days in the egg incubator in the laboratory and transferred to a table top hatching incubator with turning trays located in animal pens.

For the yolk sac inoculation, a total of 50 embryonated eggs were used. Zika virus Thailand was inoculated into 30 eggs via yolk sac on day 4 using a dose of 10^4^ PFU/ml, and 20 eggs with sterile PBS. The eggs were incubated for 11 days in the egg incubator and transferred to a table top hatching incubator in the animal pens.

Following inoculation, all eggs were assessed for viability by candling twice daily, and were allowed to hatch. Hatchability was determined and hatched chicks were evaluated for potential birth defects and abnormal clinical signs.

### RNA Extraction and Zika Virus Real-Time RT-PCR

Total RNA was extracted from allantoic fluid, yolk, whole blood and 10% tissue homogenates using MagMAx 5X Pathogen DNA/RNA kit Extraction Kit following the manufactures protocol (Applied Biosystems, USA). Two independent Zika real-time RT-PCR assays were performed to detect Zika virus RNA in the samples. Assay #1 (19) detects all known genotypes of Zika virus (FP Zika1087- 5′CCGCTGCCCAACACAAG-3′, Probe 1108FAM-5′-AGCCTACCTTGACAAGCAGTCAGACACTCAA-3′ and RP 1163c 5′-CCACTAACGTTCTTTTGCAGACAT-3′). Assay #2 which uses FP Zika4481 FP 5′-CTGTGGCATGAACCCAATAG-3′, Probe 4507cFAM 5′-CCACGCTCCAGCTGCAAAGG-3′, and RP4552c 5′-ATCCCATAGAGCACCACTCC-3′ is specific for Zika Asian genotype viruses that are currently circulating in the Western Hemisphere [CDC unpublished data, updated January 14, 2016; (20)]. All real-time RT-PCR assays were performed on an ABI 7500 Sequence Detection System (Applied Biosystems, USA). The reaction volume for the mastermix was 20 μl per sample and contained mixture 12.5 μl of 2 × Quantitec Probe master mix (Qiagen), 5.95 μl of RNase-free water, 0.25 μl Quantitect Enzyme, 0.5 μl of 100 μM of forward and reverse primers and 0.3 μl of 25 μM of TaqMan probe. To the mastermix 5 μl of extracted RNA was added, and real-time RT-PCR assays were run under the following conditions: an initial reverse transcription step at 50°C for 30 min, followed by 95°C for 15 min and 45 cycles of amplification (15 s at 94°C and 1 min at 60°C). Zika virus Thailand spiked blood and tissue samples were used as positive controls. The data were analyzed using the 7500 software V2.3 properties (Applied Biosystems, USA).

### Zika Serology

Sera were assessed for Zika virus neutralization activity using Vero cells. Animal sera from DPI 0 and DPI 21 post-inoculation were diluted 10-fold in BA-1 diluent and mixed with 200 focus-forming units of Zika virus. The virus-sera mixtures were incubated for 1 h at 37°C and transferred onto a monolayer of Vero cells in a 24-well plate at 37°C. After a 1 h incubation, the monolayers containing the virus-sera mixture were overlaid with MEM (2x) (Gibco, Life Technologies) containing 4% fetal bovine serum and 1% Noble Agar (Difco, BD). The plates were incubated at 37°C with 5% CO_2_ for 4 days and a second overlay was applied containing 1% Noble agar and 0.02% neutral red vital stain (Sigma) in MEM (2x). Plates were incubated at 37°C with 5% CO_2_ for an additional 3 days. Zika virus plaques were counted using a white light of a transilluminator (Fisher Scientific).

Immunoperoxidase staining: Presence of Zika virus specific antibodies in serum collected from infected animals were assessed by immune-peroxidase staining. Vero cells were infected with Zika virus using MOIs of 0.1, 0.01, 0.001 and incubated for 5 days at 37°C with 5% CO_2_. The medium was removed and cells were washed three times with phosphate-buffered saline (PBS)-Tween (200 ml/well) (PBST). The cells were then fixed with 10% neutral-buffered formalin in PBS (200 ml/well) for overnight at 4°C. The fixative was removed, and the cells were washed 3 times with PBS-T (200 ml/well) and blocked with blocking buffer (Sigma) for 1 h at 37°C. The blocking buffer was removed, and wells were washed three times with PBST. Sera were diluted 10-fold in PBS and 100 μl of diluted sera was added to the fixed cells and incubated for 1 h at 37°C. The primary antibody was removed and wells were washed three times with PBST. One hundred microliters of HRP-rec- Protein G antibody (Life Technologies) for sheep, pig, calf and donkey anti-chicken IgG (Jackson ImmunoResearch Laboratories) diluted 1:1,000 in PBS was added to each well for 1 h at 37°C. Plates were washed again three times with PBST and TrueBlue peroxidase substrate (KPL, Gaithersburg, MD) was added. After development, the substrate was removed and the plates were rinsed with water and dried. Plates were assessed for immunostaining by microscopy.

## Results

### Susceptibility of Sheep, Cattle, Pigs, and Chickens to Zika Infection

Following inoculation with Zika virus, sheep, cattle, pigs, and chickens did not develop any clinical signs. All infected animals were clinically normal, had physiological body temperatures throughout the study, and displayed no change in appetite. Whole blood collected at various time points from sheep, cattle, pigs and chickens was assessed for the presence Zika virus genomic material using two different real-time RT-PCR assays. Zika virus RNA was not detected in any blood sample collected at any time point following Zika virus inoculation. Three weeks after inoculation the animals were euthanized and necropsies were performed. There was no gross pathology observed during the necropsy and neural tissues (cortex, midbrain, cerebellum, and cerebrospinal fluid) from the sheep, cattle, pigs and chickens were negative for Zika virus RNA. Sheep, cattle, pigs, and chickens did not develop any detectable antibodies specific for Zika virus at any time points following inoculation by virus neutralization testing and immunoperoxidase staining.

### Susceptibility of Chicken Embryos to Zika Infection via Different Routes

**Figure 1:**
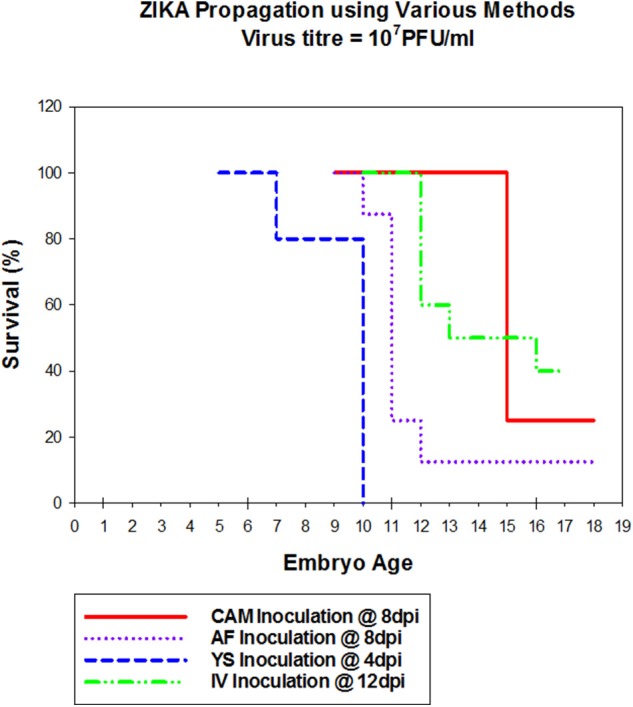
Susceptibility of Chicken embryos to Zika infection via different routes. Embryonated eggs were inoculated with 100 μl of cell culture amplified Zika virus at 10^7^pFU/ml via different routes, at different times (Cam at 8 dpi; AF at 8 dpi, YS at 4 dpi and IV at 12 dpi). Eggs were incubated at 33°C in an egg incubator and monitored daily for live embryos by candling.

**Figure 2:**
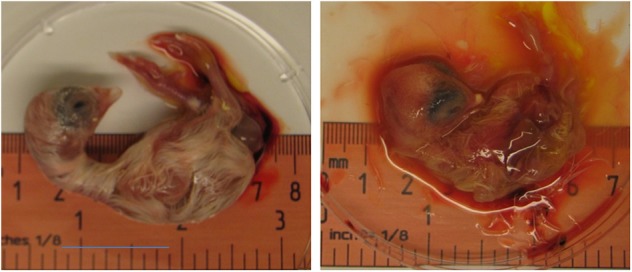
Effect of Zika virus infection on embryo development. A dead embryo from eggs inoculated with Zika virus via CAM showing malformations and hyperemia. PBS inoculated eggs were used as controls.

Zika virus Thailand strain inoculated by four different routes including IV, CAM, AF, YS were lethal to chicken embryos (Figure 1). YS inoculation of 4 day old embryos resulted in 100% mortality within 5 days, whereas AF inoculation resulted in 90% mortality within 4 days. Embryos inoculated via CAM route resulted in 80% and those inoculated via IV route resulted in 60% mortality within 7 days with no significant differences in mortality observed between the different inoculation routes. All the embryos that died following inoculation showed stunted growth and generalized hyperemia compared to PBS inoculated control embryos of same age (Figure 2). Zika genomic material was detected by real-time RT-PCR around the inoculation sites, in the brain and other tissues in embryos inoculated using the CAM and AF routes. In IV inoculated embryos, viral RNA was detected in brain and body tissues but CAM and AF samples were not tested. In yolk sac inoculated embryos, viral RNA was detected in yolk, brain and other tissues (Table 1).

**Table 1:** Detection of Zika viral RNA in tissues (CT values) of embryonated chicken following CAM, allantoic, IV, and YS inoculations.

**Route**	**Sample**	**dpi**	**CAM**	**AAF**	**Brain**	**Tissues**
**(A)**						
Chrioallantoic membrane	1	6	27.65	33.28	27.28	26.52
	2	6		31.66	27.51	31.52
	3	6		35.21	29.64	25.21
	4	6	28.37	31.56	29.11	31.97
	5	6	26.96	30.50	31.84	34.55
	6	6	28.72	34.13	32.12	34.80
Allantoic fluid	1	2	25.00	27.38	32.28	23.91
	2	2	24.14	30.83	35.23	33.69
	3	2	34.37	30.34	33.82	31.27
	4	2	27.80	28.46	30.16	28.84
	5	2	31.41	31.11	31.12	28.82
	6	3	32.69	32.15	32.20	32.22
**Route**	**Sample**	**dpi**	**Brain**	**Tissues**
**(B)**				
Intravenous	2	2	29.87	27.11
	3	2	27.19	25.83
	4	2	26.08	24.73
	5	3	25.32	27.43
	6	6	22.49	24.61
	7	7	25.14	25.64
	8	7	22.33	27.87
	9	7	24.81	27.83
**Route**	**Control**	**dpi**	**Yolk**	**Tissues**
**(C)**				
Yolk Sac	1	5	30.08	28.08
	2	5	31.50	26.12
	3	5	29.14	28.23
	4	5	34.11	29.61
	5	5	33.32	25.38
	6	5	34.77	23.25
	7	5	28.33	Path
	8	5	34.06	Path

### Determining the Optimal Dose of Zika Virus Thailand for the Hatching Experiment

**Figure 3:**
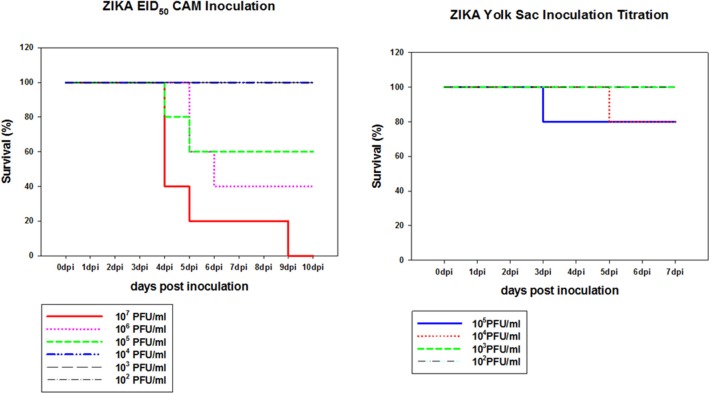
Dose titration of Zika virus Thailand by CAM and YS inoculations. Eight day old chicken embryos and 4 day old chicken embryos were inoculated with 10-fold dilutions of Zika Thailand strain via CAM and YS routes, respectively. Eggs were incubated at 33°C in an egg incubator and monitored daily for live embryos by candling.

To determine if Zika virus infection of embryonated chicken eggs will lead to congenital defects a hatching experiment was performed. Since Zika virus is lethal to chicken embryos, a dose titration was performed to determine the optimal dose which was defined as the highest dose of Zika virus Thailand that will result in less than 25% embryo deaths. This was chosen to allow for infection of the embryos without causing high levels of killing allowing embryos to hatch. Two routes of inoculation, CAM and YS, were selected for this experiment. YS route was used as it was the earliest route that could be used to infect embryonated chicken eggs. CAM route was selected for the inoculation of 8 day old embryos. IV route was not selected because it was accessible only after 11 day of embryonation so the impact of early Zika virus infection cannot be assessed. Eight day old chicken embryos inoculated via CAM route with Zika virus at 10^5^ titer resulted in 60% survival by 5 dpi and 10^6^ titer resulted in 40% survival by 6 dpi. Doses 10^4^ or less resulted in no mortality in inoculated embryos. Eggs inoculated with 10^4^ Zika virus via YS route showed 20% mortality by 5 dpi, and 80% of the embryos survived (Figure 3).

### Hatching Experiment

Detection of Zika viral RNA in tissues in embryonated chickens following yolk sac inoculation was performed in 20 day old embryos prior to hatching. Zika virus RNA was detected in the brain, eyes, heart, liver, CAM, and YS (Table 2).

**Table 2:** Detection of Zika viral RNA in tissues (CT values) of embryonated chickens and hatched chicks following yolk sac inoculation.

**Embryo**	**DO**	**DPI**	**Brain**	**Eyes**	**Heart and Liver**	**CAM**	**Yolk Sac**
**(A) EMBRYOS**							
**1**	20	15	28.93	36.43	28.24/31.89	35.07	33.45
**2**	20	15	26.81	35.29	28.73/32.85	34.29	37.15
**3**	20	15	25.06	33.94	25.03/29.67	33.15	33.10
**4**	20	15	24.73	35.31	26.52/29.47	34.90	32.68
**Chicken**	**Clinical signs**	**DPI**	**DO**	**DPH**	**Brain**	**Eyes**	**Heart**	**Liver**
**(B) ZIKA INOCULATED EMBRYOS WHICH HATCHED**							
1	None	25	30	9	No CT	No CT	No CT	No CT
6	None	25	30	9	No CT	No CT	No CT	No CT
7	None	25	30	9	No CT	35.54	No CT	No CT
10	None	25	30	9	38.18	No CT	No CT	No CT
2	Yes/found dead	18	23	2	32.66	32.04	33.66	No CT
4	Yes	18	23	2	35.44	32.23	29.91	No CT
12	Hatched small	25	30	9	36.74	No CT	37.63	No CT

**Figure 4:**
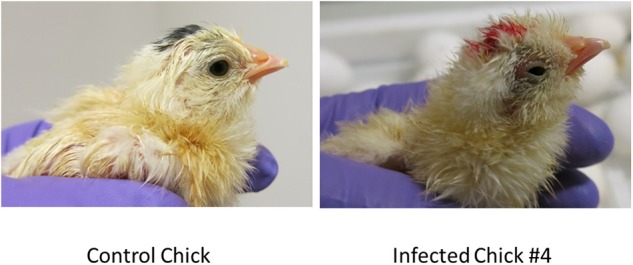
Zika virus infection can result in smaller and poorly developed chicks with neurological signs. Some chicks hatched from YS inoculated eggs showed poor development, malformations and neurological signs.

The eggs started to hatch Day 21. By day 22, 15 eggs inoculated via YS route were hatched and one of the chicks (#2) showed depression, ataxia and was sitting on hocks. Another chick (#4) was mildly depressed and ataxic (Figure 4). On day 23 chick #2 died and chick #4 started show ataxia, depression, labored breathing, and was sitting on hocks. It was euthanized the same day and assessed for Zika virus replication. Zika virus RNA was detected in the brain, eyes and heart of both chick #2 and #4 (Table 2). Fifteen eggs inoculated via YS route remained unhatched by day 25, and they were necropsied. The hatched chicks were maintained until day 30. Two additional chicks died on day 26; one exhibited crusted feathers and did not gain weight. In control group all except 4 eggs hatched and physical deformity (crooked neck and had difficulty walking straight) was only observed in one chick.

**Figure 5:**
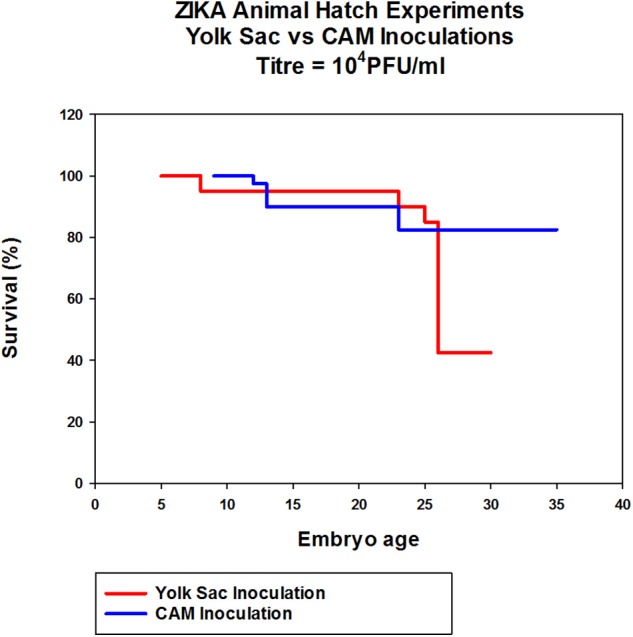
The effect of Zika virus infection on hatchability of embryonated chicken eggs. Eggs inoculated via CAM and YS were incubated for 7 days in the egg incubator and transferred to a table top hatching incubator and allowed to hatch. The eggs were candled daily throughout the experiment and the eggs with dead embryos were removed.

In eggs inoculated with Zika via CAM route showed no significant differences in hatchability and health of the chicks compared to the PBS controls. The hatchability of YS inoculated embryos was lower with only 50% eggs hatching compared to control and CAM inoculated embryos which showed over 80% hatchability although not significantly significant (Figure 5).

## Discussion

It was recently demonstrated that pig cell line PK-15 and chicken cell line DF-1 were able to support Zika virus replication (21). The ability of Zika virus to grow in these cell lines suggested that these hosts, pigs and chickens, have cells that are susceptible to Zika virus infection. The results from this study demonstrate that young sheep, cattle, pigs and chickens with an intact immune system were not susceptible to Zika virus infection. These findings are similar to another study where infectious virus was not detected in multiple species of animals common to North America, although Zika specific antibodies were detected in pigs and cottontail rabbits following experimental infection (10). The study used two different Zika virus strains, one human isolate from Cambodia in 2010, and the other isolated from a patient from Puerto Rico in 2015. The virus from Puerto Rico induced stronger neutralizing antibodies in pigs and rabbits compared to the Cambodian Zika virus eliciting antibodies at the limit of detection. This study demonstrated the influence of the Zika virus strain in the induction of antibodies in pigs (10). The Thailand Zika virus strain is greater than 98% similar to both the Cambodian and Puerto Rico strains previously used to infect animals (10).

The lack of viremia observed in sheep, cattle, pigs, and chickens demonstrate that these animals are not likely to serve as reservoirs for Zika virus. It was recently demonstrated that both rhesus and cynomologus macaques are highly susceptible to Zika infection under experimental conditions (13) and that Zika virus RNA could be detected in the brains of these animals with lymph nodes and reproductive tissue having Zika virus RNA at late stages of infection (28 days following infection). Since no Zika virus RNA was detected in neural tissues of sheep, cattle, pigs and chickens 3 weeks following infection, this is further evidence that these animal species are not susceptible hosts for Zika virus. Although viral replication in these tissues cannot be excluded at earlier times since they were not evaluated. Interestingly, fetal pigs are highly susceptible to infection during gestation (22). An additional study demonstrated that 1 day old pigs could be infected with Zika virus when inoculated by intracerebral, intradermal or intraperitoneal routes (11) indicating that neonates can still have some cells which are similar to embryonic cells with respect to permissiveness to Zika virus infection. The route of inoculation likely plays an important role for successful replication of Zika virus in neonatal pigs. Unfortunately, there have been no studies in neonatal pigs using an inoculation by infected mosquitoes. This study demonstrates that nonneonate animals are not susceptible to Zika virus infection. The biological relevance of this is that embryos of nonsusceptible animals are unlikely to become infected by a natural infection since if an infected mosquito transmitted the virus into the host, the host would not replicate the virus allowing infection of the embryo. Despite this embryos of different host animals can be infected experimentally through inoculation to serve as animal models for Zika virus infection.

It has been reported that a small percentage of cows, sheep, goats and chickens had Zika specific antibodies in sero surveillance studies (8, 9). However, it needs to be understood that the antibody detection assays used in these studies were of uncertain specificity and sensitivity. In addition, since there is cross-reaction between Zika virus and other flavivirus antibodies, this further complicates the interpretation and relevance of the data. In the experimental infections in sheep, cattle, pigs and chickens antibodies to Zika virus were not observed at 3 weeks following infection.

Even though these animal species were not susceptible to Zika virus infection, this does not mean that embryos of these species are not susceptible (11). It has been demonstrated that Zika virus can replicate in embryonated chicken eggs (23), indicating that Zika virus has a tissue tropism for embryonic tissue. Our results demonstrate that Zika virus can replicate in embryonated chicken eggs following several different inoculation routes. In addition, Zika virus can kill embryos when administered at high viral loads. These results agree with previous studies (23, 24). In addition, infection with African isolates caused higher embryo mortality compared to Asian-lineage isolates (25). In the current study, Zika virus RNA was detected in the brain, eye, heart and liver in embryos at 15 days post-inoculation. This is in agreement with a recent study where Zika virus was detected in brain, eye, heart and liver at 7 and 11 days post-inoculation into the brain vesicle in 5 day old embryos and that Zika virus suppressed chicken embryo development (25). In another study, it was also demonstrated that Zika virus infected embryonic chicken brains following injection of Zika virus into the neural tube of 2 day old chicken embryos (26). The results of Zika virus infection in chicken embryos demonstrate that Zika virus infects a broad range of tissues and cells; similar to what is observed in human infection during the first trimester of pregnancy although the cells types and tissues where Zika virus resplication occurred were different (27). Furthermore, Zika virus RNA was detected in chickens which were hatched from inoculated embryos that displayed clinical signs. In this experiment the numbers of hatched chickens that displayed clinical signs was small because it is difficult to administer a dose that will allow the embryos to hatch without killing them, and still have enough virus replication to cause birth defects similar to embryonated chicken eggs infected with high doses of Zika virus. These studies demonstrate that Zika virus has a broad tropism for chicken embryonic tissue. In addition, Zika virus has been demonstrated to be able to infect fetuses from multiple species including pigs (22), mice (16, 28), rhesus macaques (29) and humans (30). Therefore, it is likely that the embryos of sheep and cattle could possibly be susceptible to Zika virus directly inoculated, however further experiments are required to determine if this is true. It was demonstrated that mosquito eggs from Zika virus-infected mosquitoes had a slightly decreased hatch rate (31).

The results from this study confirms the study by Ragan et al. (10) that farm animals in North America are unlikely to be susceptible to Zika virus infection. Further study's using animals from different regions with different genetic backgrounds are needed to confirm that these species are not susceptible. Neonatal rabbits and pigs (10) especially neonatal pigs (11) may potentially serve as sentinel species in North America where virus is transmitted by *A. albopictus*, which will feed on these species, although further evaluation of these species as sentinels is required. The use of birds as a sentinel species as a surveillance tool for West Nile virus has been previously demonstrated (32) and is of great value in monitoring rapid global spread of zoonotic pathogens (33). The results of these studies contribute to our understanding of host tropism for Zika virus and demonstration that Zika virus has a tropism for embryonic tissue. This information is required to develop appropriate risk assessments for human and animal health.

## Data Availability Statement

All datasets generated for this study are included in the article/supplementary material.

## Ethics Statement

The animal study was reviewed and approved by Canadian Science Centre for Human and Animal Health Animal Care Committee.

## Author Contributions

AA, TT, CC-B, YB, VG, UK, DS, and SB contributed conception and design of the study. AA, TT, CC-B, YB, DS, and SB performed the experimental work. AA and SB wrote sections of the manuscript. All authors contributed to manuscript revision, read, and approved the submitted version.

### Conflict of Interest

The authors declare that the research was conducted in the absence of any commercial or financial relationships that could be construed as a potential conflict of interest.
